# Current Pretreatment/Cell Disruption and Extraction Methods Used to Improve Intracellular Lipid Recovery from Oleaginous Yeasts

**DOI:** 10.3390/microorganisms9020251

**Published:** 2021-01-27

**Authors:** Muhammad Fakhri Zainuddin, Chong Kar Fai, Arbakariya B. Ariff, Leonardo Rios-Solis, Murni Halim

**Affiliations:** 1 Department of Bioprocess Technology, Faculty of Biotechnology and Biomolecular Sciences, Universiti Putra Malaysia, Serdang 43400, Selangor, Malaysia; fakhrizainuddin8888@gmail.com (M.F.Z.); karfai.chong@hotmail.com (C.K.F.); arbarif@upm.edu.my (A.B.A.); 2 Bioprocessing and Biomanufacturing Research Centre, Faculty of Biotechnology and Biomolecular Sciences, Universiti Putra Malaysia, Serdang 43400, Selangor, Malaysia; 3 School of Engineering, Institute for Bioengineering, University of Edinburgh, Edinburgh EH9 3JL, UK; leo.rios@ed.ac.uk

**Keywords:** oleaginous yeasts, single cell oil, lipid extraction, solvent extraction, cell disruption, pretreatment

## Abstract

The production of lipids from oleaginous yeasts involves several stages starting from cultivation and lipid accumulation, biomass harvesting and finally lipids extraction. However, the complex and relatively resistant cell wall of yeasts limits the full recovery of intracellular lipids and usually solvent extraction is not sufficient to effectively extract the lipid bodies. A pretreatment or cell disruption method is hence a prerequisite prior to solvent extraction. In general, there are no recovery methods that are equally efficient for different species of oleaginous yeasts. Each method adopts different mechanisms to disrupt cells and extract the lipids, thus a systematic evaluation is essential before choosing a particular method. In this review, mechanical (bead mill, ultrasonication, homogenization and microwave) and nonmechanical (enzyme, acid, base digestions and osmotic shock) methods that are currently used for the disruption or permeabilization of oleaginous yeasts are discussed based on their principle, application and feasibility, including their effects on the lipid yield. The attempts of using conventional and “green” solvents to selectively extract lipids are compared. Other emerging methods such as automated pressurized liquid extraction, supercritical fluid extraction and simultaneous in situ lipid recovery using capturing agents are also reviewed to facilitate the choice of more effective lipid recovery methods.

## 1. Introduction

Numerous microorganisms that belong to the genera fungi, bacteria, yeast and algae have been reported to have capability to produce oils under certain cultivation conditions [1,2]. Oleaginous microorganisms are good alternative sources for industrial used oil. Depending on the fatty acid composition, the oil produced can be exploited for human consumption and in certain valuable industrial applications such as paints and coatings, detergents, cleaning products and cosmetics [3]. Moreover, the oils from oleaginous microorganisms have been increasingly explored as a substitute for high added value lipids and biodiesel feedstock due to their high lipid content, short production cycle and similar fatty acid composition to triacylglyceride from vegetable oils [4,5]. Among oleaginous microorganisms, yeast has advantages over bacteria, molds and algae. This is due to its unicellular relatively high growth rate and high lipids production ability in discrete lipid bodies [6]. Furthermore, yeast has the ability to produce “specialty-type” lipids such as cocoa butter (saturated lipids) as well as saturated exotic fats such as shea butter and sal fat that may replace the high value and expensive lipids rarely found in the plant and animal kingdom [7,8]. To date, various strategies have been developed to increase the content of intracellular saturated lipids in oleaginous yeasts [9]. Nonetheless, an efficient extraction of the intracellular lipids is one of the bottlenecks in ensuring that the use of microbial oil is commercially feasible [10].

Yeast cells are composed with thick and rigid cell walls that make the extraction of its intracellular lipid products challenging. There are no universal downstream strategies that are applicable for all yeast species and the best methods must be established and optimized for each species [11]. This is due to the fact that different yeast species may present different physical properties and cell wall structures as well as different lipid compositions [12,13,14]. A pretreatment method involving cell wall disruption after yeast cultivation is often required to make the intracellular lipids available to be extracted in the subsequent steps. For example, for the biodiesel processing that involved extraction of lipid from yeast starting from growth, harvesting, lipid extraction and lipid transesterification, the common essential step is the disruption of the cell [11,15]. Most of the work reported for lipid extraction from oleaginous yeasts describe on the dry biomass (dry route) as it allows higher recovery of lipid in comparison to the wet biomass (wet route). The extraction of lipids from the dry route has been extensively reported for analysis and small-scale research [7]. Despite the high recovery of lipids, one of the major obstacles of dry route are the high costs arising from energy and labor expenditure for the drying process prior to extraction [16]. Besides, the drying treatment may cause degradation and lipid peroxidation due to long-term exposure to high temperatures. The presence of water in wet biomass hinders the efficiency of a solvent-based lipid extraction process that may be due to limited lipid accessibility, reduced mass transfer [17] and the formation of emulsions [18]. Hence, intensive research and development is important to fully understand the mechanisms involved in the lipid extraction via wet route to allow practicality of the method at industrial scale in particular.

In general, cell disruption methods are classified according to the mechanisms they act on the cells, either by mechanical disintegration of the cells or by nonmechanical treatments via cell wall digestion or desiccation/drying at an optimized temperature [19]. The mechanical methods are further categorized as liquid (i.e., ultrasonication, microwaving, high-pressure homogenization) and solid shear methods (i.e., bead mill, grinding and freeze press) [20,21]. Meanwhile nonmechanical methods are further categorized as physical (i.e., osmotic shock, desiccation), chemical (i.e., acid, base, solvent, detergent) and biological (such as enzyme, autolysis) treatments. Practically, cell disruption is very important not only for enhancing the capability of lipid extraction, but also to evaluate the lipid content for oleaginous yeast. In general, the research trend on cell lysis of oleaginous yeast has mostly targeted for low cost with high lipid recovery to make it commercially feasible. Following cell disruption, lipids are typically extracted via liquid–liquid extraction by partitioning into organic solvents, mostly by using a combination of polar and nonpolar solvents for extraction of a greater amount of lipids. The extraction with the combination of chloroform and methanol as developed by Folch and by Bligh and Dyer, these methods are the most cited methods for lipid extractions [22,23]. To further improve the extraction of lipids, several studies have also reported on the adaptation and modifications of both methods, whereas several authors have started to investigate the usage of “green” solvents that are considered environmentally friendly in comparison to the conventional solvents such as chloroform, methanol and hexane [24].

In this review, different mechanisms of mechanical (bead milling, ultrasonication, microwave, HPH, high-speed homogenization and microwave-assisted extraction) and nonmechanical (enzyme, acid, base and osmotic shock treatments) methods are detailed and compared for a better understanding of their capability in the cell disruption and extraction action for the recovery of lipids from various oleaginous yeasts. Innovative emerging techniques such as automated pressurized liquid extraction, supercritical fluid extraction and simultaneous in situ lipid recovery using adsorbent-based oil capturing agents that were recently reported are also reviewed.

## 2. Oleaginous Yeast as Single Cell Oil

Single cell oils (SCO) are defined as edible oils obtainable from single-celled microorganisms that are primarily yeast, fungi and algae [25]. The term SCO is used in parallel to single cell protein (SCP) to represent oils of microbial origin. A small percentage of microorganisms have the ability to synthesize and accumulate 20–87% of their total biomass as intracellular lipids, which are defined as oleaginous microorganisms [26,27,28]. Furthermore, microorganisms are considered to be oleaginous if they are able to provide supply of acetyl-CoA into the cytosol, which acts as an important compound preceding for fatty acid synthetase (FAS), and as a source of NADPH, a reducing agent for fatty acid biosynthesis, respectively [29]. Throughout the fermentation process, nitrogen deprivation will trigger the lipid synthesis in oleaginous microorganisms by converting substrates such as carbon dioxide, sugars and organic acids to SCO [30]. Oleaginous microorganisms produce a wide range of lipid classes including acylglycerides, free fatty acids (FFA), phospholipids, glycolipids, lipoprotein, sterols and hydrocarbons.

De novo synthesis and ex novo syntheses are the two available routes for lipid accumulation in yeast cells [31]. Basically, for de novo lipid biosynthesis, fatty acid precursors, such as acetyl-CoA and malonyl-CoA, are generated and incorporated in the lipid storage biosynthesis. It occurs only in oleaginous microorganisms and is stimulated by nitrogen deprivation [32,33]. Hydrophilic substrates are the preferable carbon source for lipid accumulation via the de novo pathway. Carbon sources such as glucose, fructose, lactose, sucrose, whey, glucose-enriched wastes and molasses (that are categorized as sugar-based media) can be used as the substrates for de novo lipid biosynthesis [3]. During cell growth, nitrogen is consumed for cell propagation and synthesis of proteins and nucleic acid. The decrease of nitrogen content in the medium will inhibit the metabolic pathways, causing a decrease in growth rate while initiates the synthesis of fatty acids and triglycerides. Extra carbon is then directed for the synthesis of lipids. It is then accompanied by the accumulation of triglycerides in lipid bodies and within this storage period, the requirements for lipid synthesis include precursors such as acetyl-CoA, malonyl-CoA and glycerol, and energy such as ATP and NADPH. For both oleaginous and nonoleaginous microorganisms, growth rate is reduced in accordance to nitrogen content, however, for nonoleaginous microorganisms, the conversion of carbon into polysaccharides (i.e., glycogen, glucans and mananes) occurs [32]. In the meantime, several oleaginous yeasts such as *Yarrowia lipolytica*, *Geotrichum*, *Rhodosporidium*, *Cryptococcus* and *Trichosporon* are reported to survive in the hydrophobic environment and show lipid biosynthesis via ex novo synthesis [34]. In ex novo lipid biosynthesis, it is started by the introduction of fatty acid from the digestion of hydrophobic substrates into the internal environment of the cell via active transport [31]. Another way for obtaining fatty acid is by breaking down triacyglycerols or fatty esters, which is catalyzed by lipase enzyme. Then, a modification of fatty acid occurs purposely for the cell growth. The fatty acid is broken down into chain of acyl-CoAs and acetyl-CoA by enzyme acyl-CoA oxidases. Throughout this reaction, energy requirements for cell maintenance and growth are fulfilled, accompanied by organic substances that are produced as precursors for cellular materials synthesis [3]. In general, ex novo lipid biosynthesis is different from de novo lipid biosynthesis in term of nitrogen dependency. For ex novo synthesis, lipid accumulation is initiated independently from nitrogen availability in the hydrophobic medium (i.e., waste cooking oils, effluent from dairy and butter industries and industrial waste stream) and it is generated simultaneously with the cell growth [35,36].

The SCO from oleaginous microorganisms have been increasingly explored as substitutes for high added value lipids and biodiesel feedstocks [37,38,39]. SCO production by oleaginous yeasts has many advantages over bacteria, molds and algae due to their unicellular relatively fast and high growth rate, high oil content in discrete lipid bodies and the resemblance of their triacylglycerol fraction to plant oil [40]. In comparison to vegetable oils, cultivation of oleaginous yeast is not affected by climate change, seasonal production or environmental conditions, in addition to their easiness for further scaling up [14]. *Yarrowia lipolytica*, *Lipomyces lipofera*, *Lipomyces starkeyi*, *Rhodosporidium toruloides*, *Rhodotorula glutinis* and *Trichosporon oleaginosus* are among the oleaginous yeast strains that have been extensively used to accumulate oils in their cells from glucose, xylose, arabinose, mannose and glycerol [41,42,43,44]. At present, the high cost of oil production by microbial oil extraction is the major barrier to its commercialization [45]. Thus, a suitable strategy must be implemented to cater the problems from the oil production processes whilst minimizing the cost [46,47]. Among others, total production cost can be reduced by utilizing renewable feedstocks such as low-cost lignocellulosic materials and other industrial residues (containing high percentages of assimilable sugars) as fermentation substrates [48,49]. However, in an absence of cellulolytic activity in yeasts, free sugars are required to be released out from lignocellulosic materials through a pretreatment method (i.e., acid, base [50] or enzymatic hydrolysis [51]) before they can be converted into lipids by oleaginous yeasts. Furthermore, in ensuring economic viability and to minimize environmental pollution, a biorefinery approach in which intracelullar and extracellular coproducts (i.e., proteins, amino acids, carbohydrates, carotenoids, alcohols, fragrance chemicals and energy products) are valorized alongside the SCOs by oleaginous yeasts has also been suggested [48,52].

## 3. Yeast Cell Wall and Lipid Composition

Most of the lipids in oleaginous yeasts are intracellular that is stored as lipid bodies [53]. Besides lipid bodies, it is also consists of lipophilic compounds, specifically aromatic compounds that are difficult to be removed during lipid purification. The evaluation of the amount of lipid productivity can be determined by using an extraction method with organic solvents such as petroleum ether, methanol or chloroform, following the disruption of the biomass by either cellular breakage, chemical or enzymatic hydrolysis. However, many studies revealed that there is no effective method for extraction that can yield 100% of lipid from biomass [14]. Furthermore, the method would be different between yeast, algae, fungi or bacteria due to differ forms of physical properties, including the differences in cell wall and lipid compositions by different oleaginous microorganisms [21].

The composition of the yeast cell wall and its functions vary according to the species [12]. The overall structure of yeast is thicker than in Gram positive bacteria. Approximately one fourth of the dry cell weight is attributed to the thickness of the yeast cell wall, which is within the range of 100 to 200 nm [54]. Generally, yeast cell wall is mainly made up of a polysaccharide that provides strength, known as glucans, with β-(1,3) and β-(1,6) linkages. These linkages provide the yeast with a solid structure [55]. Beside glucans, it is also composed of mannans, which is a polysaccharide made up of mannose monomer linked by α-(1,6) linkage with some short oligosaccharide side chain [56]. Other constituents are also present in minimal amounts, such as chitin, proteins, lipids and inorganic phosphates [54]. Numerous bonds presented in the cell wall, especially disulphide bond, together with other components contribute to the thickness and uniqueness of the cell wall structure of yeast [12,56]. A significant change in the thickness of the cell walls can also be observed after oleaginous yeasts start to synthesize lipid droplets in their compartments [57]. The complete disruption of the wall and the release of all intracellular components requires the destruction of glucan, the main strength-providing component of the cell wall in yeast.

In general, yeast can be further divided into two categories, which are Crabtree positive yeast, and Crabtree negative yeast [30]. Under high glucose concentration, Crabtree positive yeast produces a lesser amount of lipid than that of Crabtree negative yeast due to catabolite repression. *S. cerevisiae* and *Y. lipolytica* are examples of Crabtree positive yeast, known also as respiratory deficient yeast, while respiratory sufficient yeasts such as *R. glutinis*, *C. utilis* and *Pichia stipitis* are examples of Crabtree negative yeast. Nitrogen deprivation in the medium will lead to the termination of cell division as well as the synthesis of protein and nucleic acid by yeast [1]. For nonoleaginous yeast, excess carbon is transformed into polysaccharides such as glycogen, glucan and mannan, with minimal level of lipid synthesis. However, lipid synthesis in most of the oleaginous yeast species will be stimulated during nitrogen deprivation and the product will be stored as triacylglycerides (TAG) in lipid bodies, except for *Cryptococcus terricola*, in which the lipid accumulation starts during logarithm growth, before nitrogen depletion [30].

The fatty acid composition of microbial oil is significantly affected by the types of microorganisms, substrates and culture conditions employed. Based on the fatty acid profile, microbial oils could have various applications to produce biodiesel, surfactants, waxes, lubricants and chemical feedstocks [58,59]. Oleaginous yeast has been proven to contribute to the sustainable biodiesel industry as studies showed that it can successfully accumulate microbial oil with similar composition to the conventional plant oil, which is composed mainly of oleic acid (C18:1), stearic acid (C18:0) and palmitic acid (C16:0), and low amounts of linoleic, linolenic and palmitoleic acid on substrates such as industrial glycerol, solid waste, sewage sludge or sugar cane molasses [30,60,61,62].

## 4. Pretreatment/Cell Disruption of Oleaginous Yeast to Extract Lipids

### 4.1. Mechanical Methods

Multiple methods have been developed by researchers aiming to disrupt the cell wall of the biomass. With the presence of solvents, the microbial cells usually are homogenized, but it requires force to break the cells to achieve higher lipid content within a short period of time. Mechanical methods are usually applied to crack the cells in physical ways. It is used to disintegrate the cellular structure by applying the mechanical forces or energy transfer by waves, heat or electric currents [53]. These methods have been extensively utilized for the efficient recovery of intracellular lipids from various oleaginous yeasts. Table 1 summarizes the fatty acid profiles of oleaginous yeasts grown on different substrates and extracted by different mechanical and lipid extraction methods. Mechanical disruption methods are preferably used on wet biomass because of the high cost for dewatering or drying treatment [24]. However, extraction of dry biomass is more efficient compared to wet biomass and hence it is widely used for analysis purpose on small scale research [7]. Despite the extraction being initiated from dry or wet biomass, each has its own advantages and disadvantages. Most importantly is to develop scalable mechanical lipid extraction methods with low energy consumption [63,64,65]. The comparison of energy consumption of several mechanical pretreatment/cell disruption methods quantified for various oleaginous yeasts is shown in Table 2.

#### 4.1.1. Bead Milling/Bead Beating

The working mechanism of bead beating or bead milling is to create cell disruption by physically grinding the cells by high-speed spinning against the solid surface of the fine beads during vigorous mixing [18]. The most common types of bead mill are shaking vessels and agitated beads. Shaking vessels are prevalent at laboratory scale whereby cells are ruptured by shaking the vessel on a vibrating platform. The basic structure of an agitated bead mill comprises a jacketed grinding chamber, which can either be in horizontal or vertical direction. The agitator is equipped and connected to the rotating shaft and is responsible for providing kinetic energy to the small beads inside the jacketed grinding chamber. In term of disruption and extraction efficiency, the agitated bead mill method is better than the shaking vessel bead mill. Bead milling has been successfully applied for the downstream processing of various intracellular products from laboratory to industrial scale [53]. In general, the parameters that might affect the cell rupture performance via bead milling include agitator geometry, speed, slurry flow rate, ratio of bead to substrate, biomass loading, type and diameter of bead and processing time. The impacts of bead size on processing time for lipid extraction from *Y. lipolytica* JMY5289 in form of dry and wet biomass were studied by Imatoukene et al. [24]. The experiment was conducted using a bead miller (MM400, Retsch GmbH, Germany) with stainless steel beads (sizes 2.9 and 4.9 mm) and glass beads (sizes 0.5, 1.0 and 2.0 mm) and processing times of 10, 20 and 30 s for three cycling times. Increasing the bead sizes from 0.5 to 4.9 mm increased the lipid yield regardless of the type of beads, with the lowest being 39.8% and the highest 84.7%. Likewise, increasing processing time increased the lipid extraction yield corresponding to 69.4, 78.6 and 84.7% for 10, 20 and 30 s processing time, respectively. As expected, the extraction yield of lipid was better for dry biomass compared to wet biomass.

Bead milling (using bead mill MM300 (Retsch GmbH, haan, Germany) with 425–600 µm acid washed glass beads) was demonstrated to be better at disrupting *Apiotrichum porosum* DSM 27,194 in comparison to high-pressure homogenization and ultrasonication with 74, 53 and 8% of the yeast cell disrupted, respectively [74]. In comparison to the conventional maceration method (Bligh and Dyer), bead milling extraction (performed using ULTRA-TURRAX^®^ Tube Drive (UTTD, Ika, Germany)) improved the lipid recovery from *Y. lipolytica* ATCC 20,460 by 50% corresponding to 6.82 and 13.81 g/100 g of dry weight, respectively [22]. The high centrifugal force (4000 rpm) acting on the ceramic beads for 30 min operation provoked high energy due to the shock between the beads and yeast cells, and much cellular debris was clearly viewed under scanning electron microscopy. The kinetic data obtained from this study described the performance difference between these two methods is not a limitation of time, instead the diffusion is limited due to the strong cell walls of *Y. lipolytica* (oval form of ~2 µm length and appeared as a rigid cell wall). Mechanical extraction via bead mill allowed the acceleration of dissolution and diffusion speed of targeted compounds between the matrix and the extraction solvents [75]. Another work done by Yu et al. [21] showed that bead beating with a bead beater produced a better lipid recovery from yeast, *Cryptococcus curvatus* (45.5% *w/w*) as compared to microalgae, *Chlorella sorokiniana* (6.6% *w*/*w*) due to the rigid structure of the cell wall.

#### 4.1.2. Ultrasound-Assisted Extraction

The mechanism of cell disruption via ultrasonication is linked to the cavitation phenomena, by employing ultrasound that has a frequency higher than 15–20 kHz [76,77]. Cavitation is initiated by the formation of microbubbles at nucleation sites in the fluid due to increasing acoustic power input. Then, the continuous reduction in the radius of bubbles will eventually lead to their bursting. A violent shock wave accompanied by sonic energy is then released and propagated through the medium. Mechanical energy in the form of elastic waves that resulted from the conversion of sonic energy eventually disrupts the microbial cell suspension. Ultrasonication frequency, biomass loading, extraction time and temperature are among the critical process parameters that will influence the effectiveness of microbial disruption by ultrasonication [78].

The mechanism of ultrasonication is basically targets a thinning of the yeast inner cells membranes. Such a condition was previously reported by Wu et al. [79], in which the cell wall of yeast was falling apart before affecting the cell membrane. Often, high sonic amplitudes and longer time exposure are associated with increasing of lipid recovery by sonication. In comparison with the conventional solvent extraction method, ultrasound-assisted extraction has been reported to significantly reduce extraction times, temperature and solvent volume without compromising the quality of lipids [80,81]. Jeevan Kumar and Banerjee [78] reported a better recovery of lipid from oleaginous yeast *Trichosporon* sp. with a shorter extraction time of 20 min for sonication (37% lipid yield) in comparison to Soxhlet (36% lipid yield) and binary solvent (chlororform:methanol, 2:1 (*v*/*v*)) (30% lipid yield) methods that required between 8 to 14 h. Besides, the lipids composition and fatty acid methyl esters content were of high quality that met the international standard for biodiesel. They also observed that the highest lipid content was obtained with 5 g of biomass loading while no significant increment in lipid content was observed at higher biomass loading (10 g) due to increased viscosity and surface tension that affected the cavitation process. Furthermore, using a low sonication frequency with high power (50 Hz, 2800 W) resulted in higher lipid recovery than when applying high frequency with low power (520 kHz, 40 W).

According to Wang et al. [82], a combination of chloroform–methanol (1:1, *v/v*) with assisted ultrasound was considered as a simple and effective method to increase the yield of lipid. Ultrasound has also turned out to be one of the efficient methods for lipid extraction from ascomycete *Yarrowia lipolytica* for a biojetfuel application [22]. The method also exhibited a considerably low energy consumption (51 kWh/kg microbial oil extracted) and carbon emission (45 kg CO_2_/kg microbial oil extracted) compared to maceration (175 kWh/kg microbial oil extracted and 156 CO_2_/kg microbial oil extracted) and microwave-assisted extraction processes (298 kWh/kg microbial oil extracted and 265 CO_2_/kg microbial oil extracted). Likewise, ultrasonication was reported to be the most efficient in disrupting the cell walls of *Pichia guilliermondii* in comparison to bead beater and high-speed homogenization, with 40, 35 and 12% of neutral lipid, respectively [73]. As viewed under a scanning electron microscope, cells ruptured by ultrasonication and bead beater were seen in an almost complete disruption. Meanwhile, cell-inbound-lipids, in the case of less broken cells after the disruption by high-speed homogenization, could be seen by fluorescence microscopy. In contrast, ultrasonication using Sonopuls HD 3100 (Bandelin electronic GmbH and Co. KG, Berlin, Germany) for cell disruption of *Saitozyma podzolica* DSM 27,192 and *Apiotrichum porosum* DSM 27,194 was observed to be the least disruptive in comparison to bead milling and high-pressure homogenization methods [74]. In general, ultrasonication is considered as a scalable green extraction technique that has a huge potential for efficient and rapid lipid extraction from oleaginous yeast biomass.

#### 4.1.3. High-Pressure Homogenization

Disruption of yeast through a high-pressure homogenizer (HPH) is considered one of the most environmentally safest methods. For homogenization, in the presence of positive displacement piston pump with numbers of plungers, cell disrupting procedure is initiated by directing the cell suspension into the pump cylinder through a check valve [56]. The fluid sample will eventually hit the impact ring by moving along the radial through the valve. Pressure, temperature and number of homogenization passes are among the critical process parameters that will influence the effectiveness of microbial disruption by HPH [83]. This method is widely used in pilot-scale disruption of yeast and bacteria, especially in biotechnological and pharmaceutical industries [54]. HPH is a relatively proven effective technique in rupturing yeast cell walls as compared to other mechanical techniques such as microfluidizer and bead mill [84]. HPH is also commonly applied in the industry emulsifier, and used to suspend, disperse and reduce the size of particles of products, for a better appearance, preservation of freshness and a longer shelf life [85].

Oleaginous yeasts *Saitozyma podzolica* DSM 27,192 and *A. porosum* DSM 27,194 were disrupted by HPH, EmulsiFlex-C3 (Avestin Europe GmbH, Mannheim, Germany) prior to lipid extraction using miniaturized versions of the Folch and the Bligh and Dyer methods [74]. After homogenization, cell debris was observed under microscopic imaging with 93% cells disrupted as confirmed by total cell count for *S*. *podzolica* DSM 27,192. This method, however, appeared to be less effective for *A. porosum* DSM 27,194 since only 53% cells were disrupted. This might be due to the difference in cell wall compositions and structure that are yet to be discovered for these two novel yeasts. In term of energy consumption, *A. porosum* DSM 27,194 consumed nearly twice the energy as that utilized by *S*. *podzolica* DSM 27,192 for the whole lipid extraction. Kruger et al. [86] selected a homogenizer design that has a combined feature of traditional homogenizers and microfluidizers for cell lysis of three different oleaginous yeast strains, *Lipomyces starkeyi* ATCC 12659, *Rhodotorula toruloides* DSMZ 4444 and *Cutaneotrichosporon curvatus* ATCC 20509. A maximum of 75% lipid recovery was attained under an experiment performed with a smaller orifice diameter and multiple passes (three passes as compared to single pass) that gave the highest achievable pressure (45 kpsi). Although the homogenizer managed to fully lyse the yeast cells, some lipids still could not be extracted due to the formation of emulsions that inhibited the full recovery of the hexane phase. Even though HPH might be suitable for cell lysis, it might not integrate well with the subsequent extraction protocol. Post-homogenization treatment to break the emulsions (biphasic composed of hexane and homogenized slurry) either by freezing (i.e., at 0 °C for overnight), heated (at temperatures above 100 °C for several minutes) or incubation with papain is hence recommended to improve the lipid extractability.

#### 4.1.4. High-Speed Homogenization

In this method, with the stator and rotor equipped in a high-speed homogenizer, it enables the cell suspension to be stirred at the solid–liquid interface, with high speed and shear force. With high-speed homogenization, it is economically favorable for downstream processing because it has the capability in processing high dry cell weight concentration with a short contact time. In general, high-speed homogenization increases the productivity of intracellular components extracted from the cell [77]. A high-speed benchtop homogenizer (FastPrep) was demonstrated to disrupt *R. glutinis* and *L. kononenkoae* prior to lipid extraction either by modified Folch or the Bligh and Dyer methods [87]. This type of homogenizer uses an optimized motion of multidirectional and simultaneous beating of matrix beads (bead sizes of 425–600 µm are suitable for rupture of yeast cell as recommended by equipment manufacturer) that makes it best suited for resistant cell samples. The optimum disruption parameters for *R. glutinis* was obtained when applying 4.8 shaking cycles of 47 s with 0.7 g of glass beads, followed by lipid extraction using a modified Bligh and Dyer method. In the meantime, the optimum parameters for *L. kononenkoae* were seven shaking cycles of 42 s with 0.54 g of glass beads, followed by a modified Folch method for total lipid extraction.

#### 4.1.5. Microwave-Assisted Extraction

The microwave-assisted extraction technique has been recognized as an efficient technique to extract oils from vegetable sources that markedly reduce working times, intensify the yields and preserve the quality of the extract [88,89]. Microwave energy, with a frequency of 2.45 GHz, is well known to have a significant effect on the rate of various processes in the chemical and food industry. For lipid extraction from oleaginous yeasts, electromagnetic waves are applied to the cell suspension in an organic solvent. For cell suspensions undergoing a microwave treatment, local heat is generated in dielectric or polar molecules such as water, through its friction with the electromagnetic waves at frequencies within the range of 300 MHz to 300 GHz [4,77]. Exposure of water to microwave energy will result in fast boiling, causing a rise in the internal pressure of cell and in turn, the cell expands in size. Damage caused by local heat, pressure and microwaves will cause the cell disruption, at optimal microwave frequency of 2450 MHz. In this treatment, the concentration of free water in a cell is considered as one of the parameters which determines its performance. Therefore, dilute suspension with the presence of a larger amount of water in the cell performs better in the microwave treatment because it can absorb more microwave energy for the local heating.

The feasibility of extracting lipids using microwave irradiation was previously reported by Chuck et al. [90]. A one-step method to produce fatty acid methyl esters from *R. glutanis* was developed by combining the lipid extraction in a microwave reactor with an acid-catalyzed transesterification. The rapid microwave treatment (30 s of treatment duration) did not alter the profile of fatty acid methyl esters. The advantages of using microwave technology for extraction of microbial lipids include a rapid, safe and cheap method, and dewatering of biomass sample is not required [91]. The microwave oven method was also identified as the simplest and most effective for lipid extraction from microorganisms among microwaves, sonic, heating and basic solution pretreatment [15]. *R. toruloides* Y4 that was pretreated with microwave irradiation (power setting of 800 W, frequency of 2450 ± 50 Mhz and irradiation time of 60 s) prior to enzymatic treatment obtained 62.2% lipid extraction yield that was 5.2-fold higher than that of the sample extracted without microwave treatment [91]. The microwave irradiation-treated cells appeared rough, barbed and some cells had collapsed with many holes and defects on their surface as viewed under a scanning electron microscope. Khoomrung et al. [92] invented a procedure for fast lipid extraction that allowed the extraction of lipids within 10 min. The combination of cell disruption using heating microwave and solvent extraction in one step not only reduced time for analysis, but greatly simplified sample handling and minimized sample loss during sample preparation. This method enhanced the extraction rate of lipid from *Saccharomyces cerevisiae* by 27 times. Basically, the microwave-assisted extraction used microwave energy to heat the solvent in contact with the sample and hence type and volume of solvent, processing time and temperature may directly influence the extraction efficiency [93].

Although limited studies have been reported to date, in view of its growing use for isolating organic compounds and its significant advantages, the introduction and dissemination of using microwaves for lipid extraction from oleaginous yeast seems to be acceptable for the future. Besides, microwaves can also be used to extract and transesterify the oils into biodiesel [94]. It was also reported that the recovery of biodiesel from the reaction mixture in a microwave-assisted process is approximately within 15–20 min, which is far quicker when compared to the 6 h period in the conventional heating method [95]. The main benefits are the reduction of extraction time, energy and solvent used [96]. However, the disadvantage of the microwave-assisted process is the maintenance cost involved, particularly at a commercial scale [94].

### 4.2. Nonmechanical Methods

Conventional mechanical cell disruption methods have several disadvantages, mainly long processing time and high energy consumption, and are therefore not economically feasible. Most of the energy intensive cell disruptive mechanical techniques account for up to 70% of the total production cost [97]. For instance, although bed beating, ultrasound, microwave irradiation and autoclave have been proven effective for intracellular yeast disruption, the major drawback is still the high cost for dry biomass [53,98]. The use of dry biomass will further increase the labor requirement since the dehydration process will occur within the period. However, in the wet condition, the contact between cells and solvent cannot be established due to the surface charge [99]. Hence, nonmechanical techniques such as enzyme-assisted extraction, acid and base digestions and osmotic shock treatment methods have been introduced as alternatives. The fatty acid profiles of oleaginous yeasts grown on different substrates and extracted by different nonmechanical and lipid extraction methods are tabulated in Table 3.

#### 4.2.1. Enzymatic Treatment

The use of biological methods such as enzyme treatment to disrupt yeast cell walls is a promising approach due to possible prevention of thermal degradation of lipids [7]. Enzymes can hydrolyze polysaccharide that structurally forms the cell wall and is filled with lipid bodies. Enzymatic extraction is the most preferable method for the isolation of oils from oil seeds. This method is well known in the vegetable oil industry and widely used to degrade the structures of the cell wall of the oily seed. The process is simple and easy with low energy consumption. In addition, the process is free of harmful solvents and extreme physical conditions, such as shear forces [102,103]. Nonetheless, in comparison with mechanical disruption methods, the literature reporting on utilizing enzyme-assisted extraction for oleaginous yeasts is still considerably limited.

According to Jin et al. [91], an enzymatic pretreatment method is suitable for oleaginous yeast at scalable process and well suited in the field for the development of lipid technology. They demonstrated that the use of enzyme-assisted extraction using a dialyzed solution of recombinant β-1,3-glucomannanase, plMAN5C followed by extraction with ethyl acetate resulted in an excellent lipid recovery from *R. toruloides* Y4, with 96.6% of total lipid. Later, two enzyme cocktails (first cocktail consisted of 0.025 (wt%) chitinase, mannanase and glucanase and 2.5 (wt%) papain protease from papaya; second cocktail consisted of 0.025 (wt%) chitinase, mannanase and glucanase without protease) were examined for the recovery of lipid from *Lipomyces starkeyi* [86]. The best result was obtained with the enzymatic cocktail containing papain protease that resulted in 69.3% of lipid recovery. A recent study by Heshof et al. [104] demonstrated the capability of releasing microbial oil by using an effective enzyme-assisted method for cell wall disruption of oleaginous yeast *Schwanniomyces occidentalis* CBS 2864. The tailor-made enzymes by *Trichoderma harzianum* CBS 146,429 are capable of effectively degrading the cell wall of *S. occidentalis* wet biomass after a pretreatment step using 1 M of NaOH. The enzymes composed a mixture of proteins such as proteases, amidases, glucanases, sarcosine oxidases and mannosidases. The efficiency of the yeast cell disruption was found to depend on the incubation time that was directly correlated with the concentration of enzyme. Oil was released at the highest capacity when the yeast cells were incubated with 187 mg/L of the enzyme for 4 h at pH 5 and 40 °C. Often, no further treatment was needed to be performed after the enzyme-assisted method, and it can be directly used from the cultivation broth. Hence, this improvement will bring the lipid extraction one step closer for contributing into the biobased industry with economic feasibility and viability. However, a work by Bonturi et al. [11] showed that lipid extraction by using an enzymatic pretreatment for oleaginous yeast *Lipomyces starkeyi* is not efficient due to sulfide bonds in its cell wall, which increased the strength and rigidity of its organelles. 

#### 4.2.2. Acid Treatment

The mechanism of cell disruption by acid hydrolysis is mainly targeting the release of bound lipids by dissociating lipid–starch and lipid–protein intermolecular forces [105]. Hydrochloric acid (HCl) digestion has been used as a standard lipid extraction from several oleaginous yeasts. For example, Yu et al. [21] used the HCl digestion method for the extraction of lipids from yeast *Cryptococcus curvatus* ATCC 20509. This method produced the highest lipid content of 47.30% as compared to ultrasonication and bead beating with 43.3 and 45.5%, respectively. HCl hydrolysis (1 M HCl, incubation at 60 °C for 2 h) was also found to be the most effective in disrupting the cell walls of two yeast strains, *Cyberlindnera jadinii* ATC9950 and *R. glutanis* LOCKR13 in comparison to osmotic shock, pasteurization, freezing/defrosting and homogenization with zirconia balls (0.5 mm in diameter) [106]. The amounts of lipids extracted from this acid hydrolysis pretreatment and subsequent extraction by Bligh and Dyer method (20.37 and 21.20 g/100 g biomass dry weight for *C. jadinii* and *R. glutanis*, respectively) were comparable with ultrasonication (19.53 and 17.22 g/100 g biomass dry weight for *C. jadinii* and *R. glutanis*, respectively) that was performed using Omni Ruptor 4000 homogenizer (OMNI International, Kennesaw, GA, USA). Much earlier, Zhao et al. [107] successfully digested the wet cell from *Lipomyces starkeyi* with 4 M HCl at 78 °C for 1 h before extraction with chloroform and methanol solvents. The results indicated that this oleaginous yeast responded differently to cell disruption methods in terms of lipid recovery yields. HCl digestion followed by organic solvent extraction had advantages of being able to operate at low reaction temperature and short duration time. This study also suggested that the HCl digestion method showed good adaptability to the representative yeast and could be considered as one the simplest and most effective methods for lipid extraction from oleaginous yeast. Besides HCl, sulfuric acid (H_2_SO_4_) has also proven to be effective for hydrolysis of yeast cell walls. Of the seven cell lysis approaches (bead beating, high-pressure homogenization, microwave, thermal treatments, acid, base and enzymatic treatments), acid pretreatment using 1 wt% H_2_SO_4_ (at 170 °C for 1 h) resulted in the highest lipid recovery (91.5%) from *L. starkeyi* [86]. The same acid pretreatment condition was found to be equally efficient for two other oleaginous yeasts, *Cutaneotrichosporon curvatus* (99.7%) and *R. toruloides* (88.5%). The preliminary scale-up of acid pretreatment (from 4 to 300 mL using a steam-heated reactor to mimic the industrial scale) using higher wet loading of *R. toruloides* biomass with slightly lower temperatures or shorter pretreatments time had resulted in higher lipid yields than those obtained for the small scale. This work also demonstrates the effectiveness of H_2_SO_4_ in the recovery of lipids from yeasts, which may enable less large-scale reactor metallurgy as compared to HCL.

#### 4.2.3. Base Treatment

Similarly, like acid treatment, base treatment is often selected to catalyze the hydrolysis of polysaccharides and proteins. However, in comparison with acid, base treatment is far less effective and hence it is rarely used for lipid recovery from oleaginous yeasts. When comparing the acid (H_2_SO_4_) and base (NaOH) treatments for lipids recovery from *L. starkeyi*, Kruger et al. [86] found that base treatment failed to recover the intracellular lipids, with recovery percentage of <1% in contrast to acid treatment that successfully recovered the lipids with 91.5%. The failure is likely due to the formation of a gel from the residual solids that resulted in poor agitation in the subsequent step involving hexane extraction. At high pH, the gel might be formed due to deprotonation or denaturation of cell walls that changed the surface charges.

#### 4.2.4. Osmotic Shock Treatment

For the osmotic shock treatment, high osmotic pressure is applied on the cell when it is place in a medium with high concentration of solute. It is then followed by diluting the medium, forming a water-rich environment. Due to the difference in osmotic pressure, movement of water molecules into the cell can be observed and this will result in a rise of intracellular pressure that in turn reduces the strength of cell wall instead of thoroughly disrupting it; [4,106] recently utilized osmotic shock for the disruption of two oleaginous yeasts, *Cyberlindnera jadinii* ATCC 9950 and *R. glutinis* LOCKR13. The procedure involved the dried yeast cell biomass being suspended in 10% (*w*/*v*) NaCl solution and incubated for 48 h at room temperature. In comparison, a higher total lipid content was extracted for *R. glutinis* (14.10 g/100 g dry weight) than *C. jadinii* (11.93 g/100 g dry weight). The lipid content in *C. jadinii* obtained from the osmotic shock treatment was the lowest in comparison to acid hydrolysis, sonication, homogenization, freezing/defrosting and pasteurization methods. Meanwhile, the lipid content in *R. glutinis* obtained from osmotic shock treatment was slightly higher than that observed for homogenization and pasteurization methods but lower than the acid hydrolysis, sonication, and freezing/defrosting methods. The recovered lipids were found to be similar to the composition of fatty acids (oleic acid (44–48%), palmitic (17–18%), stearic (15–18%), behenic (2–5%), linoleic (2–4%), arahinic (0.9–3%) and other fatty acids (less than 1%)) obtained using pasteurization and alternating freezing/defrosting methods. In comparison with oleaginous algae [108,109], only a limited number of recent studies reported on utilizing osmotic shock for the disruption of oleaginous yeasts. Even though osmotic shock is one of the simplest pretreatment methods for the extraction of intracellular lipids, it only showed low or mild rupture effect and required a considerably long duration of treatment. To date, there is no large scale application of an osmotic shock for lipids extraction due to the usage of large volumes of water and the requirement for an efficient cooling system, which results in a high total cost of operation [110,111].

## 5. Total Lipid Extraction Methods

Most microbial lipid extraction methods generally involve a cell rupture method followed by an organic solvent extraction. Various organic solvents have been suggested to selectively extract lipids from oleaginous yeasts. Soxhlet extraction, Bligh and Dyer, and Folch are the three classical methods developed for total lipid extraction that are extensively used currently. Rapid and easy processing are the major advantages of these methods; however, they are less sensitive when compared with other new emerging methods.

### 5.1. Soxhlet Extraction

Soxhlet extraction is traditional and known as a standard semicontinuous method that is usually applied for extracting lipids from food products [112]. For extracting organic solutes such as lipids, an organic solvent such as hexane is commonly used as the extractant [113]. The solvent is vaporized in a round bottom flask and the condensed solvent (in a condenser) is allowed to percolate through the powdered biomass before refluxing into the flask. After several cycles of evaporation, condensation and percolation of solvent through the biomass sample, the flask containing a mixture of solvent and extracted lipids is taken out to recover the crude lipids after vaporizing the solvent using a rota-evaporator [114]. Dalmas Neto et al. [115] evaluated the performance of three different solvents, hexane, chloroform and chloroform/methanol (2:1), for the extraction of lipids from dried *R. toruloides* DEBB 5533 using Soxhlet extraction. They discovered the amounts of lipids obtained were comparable with 46.1, 44.7 and 43.2% for hexane, chloroform/methanol and chloroform, respectively. Meanwhile, the efficiency of polar solvents such as ethanol was found to be higher than that observed for nonpolar solvents, such as hexane, in extracting polar lipids [116]. In comparison with a modified Bligh and Dyer method (using methanol and chloroform (2:1 *v/v*)), the Soxhlet extraction (using the same solvent mixture with a frequency of 5 cycles) was found to be less efficient in extracting lipids from *R. glutanis*, with 98 and 50% extraction efficiency, respectively [117]. The disadvantage of long operation time (usually around 8 h) of conventional Soxhlet extraction can be overcome by combining the Soxhlet extraction with microwave heating (operation time can be reduced to less than 1 h) [118].

### 5.2. Bligh and Dyer Method

The classical Bligh and Dyer method is considered as a standard method, the most cited and extensively used for total extraction of lipids from oleaginous yeasts. The method uses the combination of different solvents consisting of chloroform:methanol:water (1:2:0.25, *v*/*v*/*v*) [119]. The monophasic ternary system is converted into a biphasic state by dilution with additional chloroform and water. This combination of solvents and water in this ratio formed a separation into two phases, with the lower (chloroform) phase containing all the extracted lipids. Instead of using water, some researchers used 1 M NaCl in order to prevent binding of acidic lipids to denatured lipids [94]. Another report suggested that the addition of 5% trichloroacetic acid during extraction, which resulted in a six-fold increase of lipid yield compared to the classical Bligh and Dyer method [120].

### 5.3. Folch Method

The Folch method is another lipid extraction method known for its efficiency in lipid recovery at laboratory scale. The method is almost identical to Bligh and Dyer method, but uses a different ratio of solvents, chloroform:methanol (2:1 *v/v*), and the protocol includes washing the extract by the addition of a certain amount of salt solution. The mixture of solvents and cells resulted in a separation of two layers, consisting of pure lipid in lower phase and organic solvents in the upper phase [121]. Table 4 summarizes lipid recovery from various oleaginous yeasts extracted by the Folch and the Bligh and Dyer methods after a pretreatment either using a mechanical or nonmechanical method.

### 5.4. Extraction with a Combination of Different Solvents

An efficient extraction requires that the solvent fully penetrates the cell mass with a polarity similar to that of the target compounds [128]. Yeasts characteristic of having a dense cell wall, which can be recalcitrant to many solvents, can make the extraction of the intracellularly stored lipid become more challenging [11]. Besides extraction efficiency and easy separability, the best criteria for selecting the proper solvents for lipid extraction method include selectivity and nonreactivity of the solvents with the lipids of interest. According to Vasconcelos et al. [87], the combinations of chloroform and methanol are the most utilized methods for extraction of lipids from oleaginous yeasts, despite the toxicity of both solvents. To date, various adaptations and modifications of the famous Bligh and Dyer and the Folch methods have been employed, including varying the solvent ratios and using different combinations of solvents consisting of polar and nonpolar solvents, mainly aiming to further enhance the lipid extractions. For example, Lewis et al. [129] demonstrated the extraction of lipid from microheterotrophs by using a combination of biphasic solvent system, hexane: chloroform with a ratio of 4:1. About 96.6% of the total lipids were managed to be extracted from *R. toruloides* by using an ethyl acetate as the substitute for chloroform [91]. The protocol was conducted at room temperature for 1 h without dewatering. Furthermore, Vasconcelos et al. [87] adapted the modified Bligh and Dyer method for the optimization of lipid extraction from two oleaginous yeasts *R. glutanis* and *Lipomyces kononenkoae*. After cell disruption by using a high-speed homogenizer (FastPrep), lipids were extracted by several combinations of two different solvents (chloroform:methanol; chloroform:ethanol; tolune:methanol; tolune:ethanol) and K_2_HPO_4_ buffer in the ratio of 1:1:0.9. The combination of toluene: methanol: K_2_HPO_4_ was the best extraction solvent for *L. kononenkoae*, while chloroform: methanol: K_2_HPO_4_ excelled for *R. glutinis*, with the highest percentage of lipids obtained being 17.9 and 15.7%, respectively. The higher lipid extracted in toluene compared to chloroform is a good indication for substituting the chloroform from the Bligh and Dyer method with less harmful solvents for mitigating impact on health and the environment. 

### 5.5. “Green” Solvent for Lipid Recovery

Petroleum solvents such as hexane and a mixture of hydrocarbons are among the commonly used solvents for the extraction of lipids from oleaginous microorganisms [130]. Hexane is widely used for lipid extraction because it has excellent solubilizing ability and narrow boiling points (between 63–69 °C) [131]. However, when inhaled by humans, hexane may affect the neural system [132]. During extraction, hexane is released into the environment and reacts with the pollutants to form ozone and photochemicals [130]. Meanwhile, chloroform, which is used as a solvent in Bligh and Dyer and the Folch methods, is a carcinogenic chemical and classified as a hazardous air pollutant (HAP) in the United State and hence requires extra care in its handling operation. Some chemists suggest dichloromethane as the substitute for chloroform, but it is also considered as HAP and prohibitive for use in a large-scale volume [133]. Currently, health, safety and environmental concerns have triggered the search for “green” solvents as the substitute for these solvents without compromising the lipids yield.

To replace the conventional solvent, n-hexane, Imatoukene et al. [24] investigated the use of several “green” solvents that are known as environmentally friendly, such as terpenes (*p*-cymene and *d*-limonene) and esters (isoamyl acetate, butyl acetate and ethyl acetate) for the extraction of lipids from *Y. lipolytica* JMY5289. Among the “green” solvents, the highest lipid yield was obtained by isoamyl acetate. Further, the proportion of saturated and unsaturated fatty acids acquired was almost identical with n-hexane. In the meantime, the lowest lipid extraction yield from *Y. lipolytica* JMY5289 was obtained by ethanol, the cheapest “green” solvent in comparison to the other solvents tested. This inefficient extraction might be due to the polar nature of ethanol that contrasts with the lipids in *Y. lipolytica*, which are neutral [134,135]. The insignificant differences between esters and terpenes solvents with n-hexane for the lipids extraction hence highlighted the potential of these “green” solvents as the substitutes for the conventional solvents [136,137]. Based on the screening of ten different “green” biobased solvents, Breil et al. [138] found that cyclopentyl methyl ether, 2-methyltetrahydrofuran and ethyl acetate were among the good candidates for replacing hexane during lipids extraction from *Y. lipolytica* IFP29. Their fatty acids profiles (mostly oleic acid (C18:1), linoleic acid (C18:2n6) and palmitic acid (C16:0) with minor amounts of palmitoleic acid (C16:1) and stearic acids) were shown to be similar to hexane in terms of selectivity. However, they discovered p-cymene, *d*-Limonene, α-pinene, ethyl lactate, isopropanol and ethanol were not suitable for replacing hexane, mainly due technical and economic reasons.

In searching for a greener alternative to chloroform, cyclopentyl methyl ether (CPME) was evaluated for its capability in extracting oils or triacylglyderol from wet cells of *Lipomyces starkeyi* ATCC 56,304 [139]. The biphasic system of CPME:methanol:water (using a starting ratio of 1:1.7:06 and a final ratio of 1:1:08) resulted in triacylglyderol extraction efficiency that was comparable to Bligh and Dyer method (chloroform:methanol:water) with 64.6 and 66%, respectively. However, a monophasic system of CPME:water (using a ratio of 1:0.7) performed poorly, having the lowest triacylglyderol extraction efficiency in comparison to hexane:water, chloroform:water and Bligh and Dyer method. Regardless, the fatty acid profile remained constant for all solvent systems.

Further, Yook et al. [140] investigated the effectiveness of selected switchable hydrophilicity solvents (SHS) towards yeast strain, *Y. lipolytica*. The solvents, namely N-ethylbutylamine, N-dipropylamine and N, N-dimethylcyclohexyl-amine, were used for the extractions. In general, SHS is a green solvent in which its hydrophilic and hydrophobic properties can reversibly change upon CO_2_ entry and removal (by heating and/or sparging the mixture with air). In comparison with the mixture of conventional organic solvents (chloroform/methanol), N, N-dimethylcyclohexyl-amine and N-ethylbutylamine managed to increase the lipids yield by 13%. It was also found that their fatty acid compositions were comparable to those obtained with the conventional solvents, thus assuring the quality of biodiesel produced through this alternative method. In addition, this study also proved the capability of lipid extraction using SHS that could significantly improve the energy efficiency and economic feasibility of an oleaginous yeast-based biodiesel production process. Furthermore, SHS fulfills the concept of green chemistry for the development of efficient, nonwasteful and environmentally friendly separation technology [141].

## 6. Other Emerging Methods

Recently, an automated pressurized liquid extraction method (APLE) was reported for the first time for the extraction of lipid from dried cells of oleaginous strains [142]. APLE is a simple procedure that employs elevated temperatures and pressures to achieve extraction within a short period of time. APLE is commonly utilized for the extraction of environmental biotic samples [143] and some also reported on its potential application for the extraction of bioactive compounds from biomass cells [144,145]. The application of APLE system (i.e., APLE-3500, Titan Instruments Co., Ltd., Beijing, Chine) for the extraction of intracellular lipids as recently described by Li et al. [142], basically involved several main steps: (i) mixing the lyophilized yeast cells with diatomite in a mortar; (ii) transferring the yeast cells into a stainless steel chamber through a funnel; (iii) placing chamber in the APLE system that equipped with solvents (i.e., chloroform and methanol in the ratio of 1:1) and collection bottles; and (iv) setting the extraction parameters (i.e., temperature, pressure and operation time). Temperature effected the lipid extraction yield with the optimum obtained at 105 °C with around 89% lipids extraction yield for both *R. toruloides* CGMCC 2.1389 and *Cryptococcus curvatus* ATCC 20509. In terms of lipid static extraction time, *R. toruloides* CGMCC 2.1389 required 40 min to achieve 95.2% lipid extraction yield, while *C. curvatus* ATCC 20,509 needed only 15 min to reach its optimum yield of 89.5%. However, prolonging the extraction time to 20 min decreased the yield to 83.8%. Furthermore, the fatty acid compositions of the neutral lipids of both yeasts obtained from the APLE system were identical to the profile obtained with the conventional acid–heating extraction method. This result thus emphasized the potential of APLE to be used as an efficient lipid extraction method, particularly for oleaginous yeasts in a short period of operating time and without the need for harsh pretreatment procedures.

Supercritical fluid extraction (SFE) has emerged as an alternative method to traditional methods of using organic solvents in liquid–liquid extraction for the extraction and fractionation of various bioactive compounds. However, to date, there are still limited reports describing the utilization of SFE for yeast. Carbon dioxide (CO_2_) that is commonly used for SFE offers several advantages, such as safe, nontoxic and inexpensive, compared to other conventional solvents [146,147]. Besides, CO_2_ is also easily separated after the process. In order to maximize the extraction yield, SFE can be modified by manipulating the process variables (i.e., temperature, pressure, flow rate and bed size) and use in a combination with other extraction techniques such as ultrasonication [148] and different types of cosolvents [146]. SFE is typically inefficient without cell disruption. Mechanical or thermal cell wall disruption was reported to improve the microbial lipid extraction via SFE employing CO_2_ [122,149], demonstrated on a recovery of lipids from the yeast, *Candida* sp. LEB-M3, by combining SFE with an ultrasound-assisted unit. The lipid extraction was carried out under conditions that ensured CO_2_ was in a supercritical state while the ultrasonic wave helped to increase the contact between matrix and CO_2_. In comparison with the conventional Bligh and Dyer method, the lipid yield from SFE with the ultrasound-assisted unit was considerably low. However, SFE was a highly selective method to obtain nonpolar components by controlling the temperature and pressure. This makes it a potential method with operational advantages, such as the recovery of lipids of interest that is not accompanied by polar lipids and other impurity compounds that otherwise need to be removed by complex downstream processing operations. Earlier, Milanesio et al. [145], who investigated on lipids extraction from *Y. lipolytica*, also came to a conclusion that the supercritical CO_2_ required an efficient cell disruption method to improve the yield of lipid. Despite a lower lipid yield in comparison to conventional liquid–liquid extraction methods, SFE in combination with an effective cell disruption method could still be devised as a promising environmentally friendly alternative. Nevertheless, at industrial scale, further research and development is vital to scale up the system as the combination of high-pressure equipment with dry cells would lead to high operating cost [150].

Another promising lipid recovery system is by utilizing adsorbent-based oil capturing agents for simultaneous in situ lipid recovery during fermentation. This system was recently employed for the lipid recovery from *Y. lipolytica* NCIM 3590 [151]. Adsorption resin, SEPABEADS^TM^ SP70 (Mitsubishi Chemical Corporation, India) was used as the oil-capturing agent that was introduced in the fermentation broth. For a comparison, the oil produced from *Y. lipolytica* cells was extracted in the form of intracellular lipid, extracellular lipid (in the fermentation broth) and as an adsorbed oil on resins. The glucose uptake rate was increased in the presence of the oil-capturing agent, suggesting that the removal of lipid droplets from cell surfaces leads to glucose assimilation and flux redirection for the synthesis of triacylglycerols. The on-line oil capture over an adsorbent bed fluidized with fermentation broth resulted in lipid content higher than 89%. This system allowed for continuous production and lipid recovery that could be operated for more than 380 h. This in situ oil adsorptive fermentation system allowed for repeated cell use for extended periods that demonstrated an effective integration of upstream and downstream processing that is industrially feasible. Furthermore, the fatty acid composition of the resins-adsorbed oil was comparably different from the intracellular oil (extracted in the fermentation broth without the presence of adsorbent resins). However, based on its improved fatty acid compositions, it is estimated that the biofuel properties of the fatty acids extracted by the resin-adsorbed oil would produce smoother engine operation and have a better performance at lower temperature with reduced emissions that are competent to plant-based oils [152].

## 7. Summary and Conclusions

This review article summarized the methods for pretreatment or cell wall disruption followed by extractions of intracellular lipids from various oleaginous yeasts. An effective cell disruption is a vital step towards an effective extraction of lipids from most yeasts. Multiple mechanical and nonmechanical methods have been developed to disrupt the cell wall of oleaginous yeasts. These methods can be used on dry or wet biomass, but whenever applicable wet biomass is preferred as it will eliminate the cost of drying.

Mechanical methods employed for lipid extraction are highly scalable but usually consume high amounts of energy. Depending on the yeast strains, these methods are reported to be better from one another at extracting intracellular lipids. The variables that influence the cell wall rupture in yeasts should be studied, aiming at greater recovery and quality of the extracted lipids. Moreover, understanding the mechanisms behind the cell disruption is important in finding the most applicable and practical method for different oleaginous yeast species. Bead milling can eliminate the drying biomass step and hence reduce the overall production cost. Ultrasonication is considered as a simple and green method that can be operated at mild temperatures and pressures with reduced time without compromising on the lipid quality. Microwave-assisted extraction is one of the simplest and effective methods for lipid extraction; however, it requires high electricity costs that are not economically feasible at industrial scale. High-pressure homogenization has an established track record for an industrial scale and does not require chemical reagents. Although nonmechanical methods of cell disruption require less energy than the mechanical methods, their application is often restricted to small-scale operation mainly because of favorable economic conditions. Enzymatic treatment may be potentially beneficial as it can decrease the operating cost if it is successfully immobilized to facilitate its recovery and reusability. The use of acid digestion can be considered as one the simplest and most effective methods for lipid extraction from oleaginous yeast. Nonetheless, the usage of chemicals for lipid extraction is considered unsustainable because of challenges in the scale-up stages. In general, to achieve an optimal recovery yield of lipid, further studies must focus on the optimization of operational parameters and may also consider the combination of the mechanical and nonmechanical methods.

Following cell disruption, intracellular lipids are extracted via solvent extraction. The combinations of chloroform, hexane and methanol are the most used solvents for lipid extraction from oleaginous yeasts. The potential of using “green” solvents in comparison to these conventional solvents has been demonstrated for several oleaginous yeasts with comparable extraction performance and no significant impact on the composition of lipids. To accurately evaluate the productivity of lipids in yeasts, it is essential to use an extraction method that is efficient and reproducible. Emerging methods such as supercritical fluid extraction using CO_2_ is considered as an efficient, mild and “green” alternative method for lipid extraction. Meanwhile, novel methods such as automated pressurized liquid extraction and simultaneous in situ lipid recovery using adsorbent-based oil capturing agents are promising methods that allow for an efficient recovery of lipid from oleaginous yeasts in a short period of operating time and without the need for harsh pretreatment procedures.

Overall, the study of energy consumption and environmental impact will help to determine which methods are the best alternatives for lipids recovery from different yeast species. In addition, kinetic extraction studies will further explain whether the ruptured of the yeast cell wall is the factor that permits the enhancement of mass transfer of lipids from biomass to solvent. In addition, it is important to keep in mind the possibility of scale-up and economic viability of the technique.

## Figures and Tables

**Table 1:** Fatty acid profile of oleaginous yeasts extracted using various mechanical pretreatment/cell disruption and lipid extraction methods.

Oleaginous Yeast	Fermentation Medium	Mechanical Pretreatment	Lipid Extraction Method	Fatty Acid Profile (%)											Reference
				C12:0	C14:0	C16:0	C16:1	C17:0	C18:0	C18:1	C18:2	C18:3	C20:0	C24:0	
*Trichosporon mycotoxinivorans* S2	Paddy straw	Ultrasonic homogenizer	chloroform and methanol (2:1 *v*/*v*)	5.36	−	18.28	7.60	−	18.64	30.84	2.36	−	−	−	[66]
*Naganishia liquefaciens* NITTS2	Municipal waste activated sludge	Sonication	Chloroform: methanol at (1:1 *v*/*v*)	−	0.6	23.1	-	−	14.2	38.4	21.4	1.1	−	−	[67]
*Yarrowia lipolytica* JMY5578	Yeast extract, dextrose, ammonium chloride and monopotassium phosphate	High voltage electrical discharges	2 mL of KCl (1 M): methanol solution (4:1, *v*/*v*, with 0.034% MgCl_2_)	−	0.07	22.57	−	2.24	6.91	17.94	0.34	17.68	0.62	2.18	[68]
*Cryptococcus vishniaccii* MTCC 232	Paper mill sludge extract	Sonicator	Modified Bligh and Dyer method	−	0.18 ± 0.07	24.59 ± 0.45		−	1.66 ± 0.32	44.50 ± 0.41	8.60 ± 0.34	0.02 ± 0.01	2.64 ± 0.09	−	[69]
*Cryptococcus curvatus* MUCL 29819	Acetic Acid	Beads beating	Chloroform:methanol (1:1, *v*/*v*)	−	−	8.38		0.40	29.75	50.37	6.44	1.50	−	−	[70]
*Yarrowia lipolytica* TISTR 5151	Palm oil mill effluent with gylcerol	Sonicator	Chloroform:methanol (2:1, *v*/*v*)	−	0.60	41.53		−	7.09	36.99	11.90	0.44	−	0.22	[71]
*Cryptococcus curvatus* ATCC 20509	YPD media	Autoclaving	Chloroform:methanol (1:1, *v*/*v*)	−	0.9 ± 0.1	29.4 ± 1.0		−	18.6 ± 0.8	44.9 ± 2.9	5.0 ± 0.3	−	−	−	[21]
		Microwave		−	0.7 ± 0.0	31.5 ± 2.0		−	18.8 ± 1.1	47.6 ± 3.3	0.3 ± 0.0	−	−	−	
		Sonication		−	0.4 ± 0.0	27.0 ± 1.3		−	18.3 ± 0.8	47.5 ± 3.7	5.6 ± 0.4	0.4 ± 0.0	−	−	
		Bead beating		−	0.6 ± 0.0	27.4 ± 2.0		−	17.8 ± 0.6	46.8 ± 2.3	6.1 ± 0.4	0.5 ± 0.1	−	−	
*Saccharomyces cerevisiae* CEN.PK113-7D	Nitrogen-limited media	Microwave-assisted methods	Chloroform:methanol (1:1 *v*/*v*)	−	−	13.7 ± 0.3		−	3.5 ± 0.3	30.0 ± 0.5	−	−	−	−	[72]
*Trichosporon oleaginosus* DSM11815				−	−	29.7 ± 1.1		−	6.4 ± 0.1	48.3 ± 0	9.8 ± 0.8	0.3 ± 0	−	−	
*Rhodotorula graminis* DSM 27356				−	−	22.8 ± 0.2		−	1.8 ± 0	46.9 ± 0.3	1.88 ± 1.0	2.0 ± 0	−	−	
*Lipomyces Starkeyi* DSM 70296				−	−	31.6 ± 0.2		−	10.1 ± 0	47.8 ± 0.3	4.2 ± 0.8	0.1 ± 0	−	−	
*Rhodosporidium Toruloides* DSM 70398				−	−	27.7 ± 0.9		−	14.6 ± 1.2	36.6 ± 1.7	14.7 ± 1.4	1.8 ± 0.1	−	−	
*Yarrowia lipolytica* CBS 6124				−	−	13.6 ± 0.1		−	2.1 ± 0.2	46.2 ± 0.5	11.2 ± 0.8	−	−	−	

**Table 2:** Comparison of energy consumption by mechanical cell disruption methods for the extraction of lipids from oleaginous yeasts.

Yeast Strain	Method	Experimental Parameter	Energy Consumption (kWh/kg Oil)	Carbon Emission (kg CO_2_/kg Oil)	Lipid Yield (%)	Reference
*Yarrowia lipolytica* ATCC 20460	Ultrasound	20 °C, 30 min	51	45	8.10	[22]
	Bead milling	20 °C, 30 min	32	28	13.16	
	Microwave	110 °C, 30 min	54	48	8.18	
*Pichia guilliermondii*	Ultrasound	10 min (45 s On, 15 s Off), 125 W	42.5	−	40	[73]
	Bead milling	0.2 mm glass bead, 851 W	170	−	35	
	High-pressure homogenization	20,000 rpm, 500 W	289	−	12	
*Saitozyma podzolica* DSM 27,192	Ultrasonic	60 °C, 20 min (50 s On, 10 s Off)	107	−	20.7	[74]
	Bead mill	425–600 µm glass beads, 60 °C, 20 min	110	−	32.3	
	High-pressure homogenization	60 °C, 20 min	102	−	43.4	
*Apiotrichum porosum* DSM 27194	Bead mill	425–600 µm glass beads, 60 °C, 20 min	161	−	20.0	[74]
	High-pressure homogenization	60 °C, 20 min	283	−	14.1	

**Table 3:** Fatty acid profile of oleaginous yeasts extracted using various nonmechanical pretreatment/cell disruption and total lipid extraction methods.

Oleaginous Yeast	Fermentation Medium	Non-Mechanical Pretreatment	Lipid Extraction Method	Fatty Acid Profile (%)									Reference
				C14:0	C16:0	C17:0	C18:0	C18:1	C18:2	C18:3	C20:0	C24:0	
*Rhodotorula glutinis* NRRL YB-252	Raw glycerol	Treated with 2 M HCl	Bligh and Dyer method	−	20.4	−	−	1.1	68.3	4.0	−	−	[100]
*Lipomyces starkeyi* NRRL Y-11557				−	39.3	−	−	7.8	42.5	4.2	−	−	
*Lipomyces lipofer* NRRL Y-11555				−	35.1	−	−	8.2	43.1	3.7	−	−	
*Cryptococcus curvatus* NRRL Y-1511				−	17.3	−	−	18.0	49.5	10.6	1.7	−	
*Candida cylindracea* NRRL Y-17506				−	30.0	−	−	7.3	29.3	23.1	3.3	−	
*Rhodosporidium toruloides* DEBB 5533	Sugarcane and urea	Mineral acids	Solvent recovery chloroform and sample, 1:1	1.0	21.5	0.0	4.6	62.1	7.6	0.7	0.1	0.7	[101]
*Cryptococcus curvatus* ATCC 20509	YPD media	HCl digestion	Chloroform and methanol (1:1, *v*/*v*)	0.5 ± 0.1	26.6 ± 1.4	−	18.2 ± 0.9	47.3 ± 3.4	5.9 ± 0.3	0.5 ± 0.0	−	−	[21]

**Table 4:** Applications of the Bligh and Dyer and the Folch methods for the recovery of intracellular lipids from various oleaginous yeasts.

Total Extraction Method	Yeast Strain	Pretreatment/Cell Disruption Method	Experimental Parameters/Conditions	Lipid Yield	Lipid Productivity (%)	Reference
**Bligh and Dyer Method**	*Rhodotorula glutinis*	Bead beating	8 cycles of 45 s with 0.5 g of glass beads	−	23.5	[87]
	*Candida sp.* LEB-M3	Supercritical fluid assisted by ultrasound	45 min ultrasound, 800 W of power	−	32.75 ± 3.01	[122]
	*Rhodosporidium kratochvilovae* HIMPA 1	Ultrasonication and microwave treatment	2 min of sonication, 40 KHz of power, followed by 90 °C temperature in microwave for 15 min	−	47.13	[123]
	*Pichia guilliermondii*	Bead beating	10 min in bead beater, loaded with 0.2 mm glass beads	−	35	[73]
		Sonication	10 min sonication, 125 W of power in pulse mode (45 s On; 15 s off) at 50% amplitude		40	
		Homogenization	Homogenizer shaft rotating at 20,000 rpm with on-off, at 500 W of power		12	
	*Trichosporan sp.*	Sonication	20 min sonication, at 30 °C with a frequency of 50 Hz and 2800 W power	−	43 ± 0.33	[78]
	*Lipomyces starkeyi* ATCC 56304	Sonication and Fenton’s reagent	10 min sonicator at a frequency of 35 kHz	1.62 g/L	21.32 ± 0.82	[124]
	*Yarrowia lipolytica* IFP29 ATCC 20460	Ultrasonic-assisted extraction	30 min sonication, frequency 20 kHz with 300 W of power	8.10 ± 0.24 g/100 g dw	−	[22]
		Bead milling	4000 rpm for 30 min, using 20 g of ceramic beads	12.73 ± 0.41 g/100 g dw		
		Microwave-assisted extraction	1000 W of power and a temperature at 110 °C	7.13 ± 0.45 g/100 g dw		
	*Cyberlindnera jadinii* ATCC 9950	Acid hydrolysis	10 mL of 1 M HCl and incubated for 2 h in a 60 °C	20.37 ± 1.20 g/100 g dw	−	[106]
		Sonication	Frequency 20 kHz for 3 min of operation and 2 min of break (2 cycles)	19.53 ±1.59 g/100 g dw		
		Osmotic shock	10% (*w/v*) NaCl solution, stirred for 1 min, left for 48 h	11.93 ±1.67 g/100 g dw		
		Pasteurization	Placed in water bath at 80 °C for 30 min	13.31 ±0.65 g/100 g dw		
		Homogenization with zirconium beads	1 g of 0.5 mm diameter beads and mixed for 30 min	15.26 ±0.94 g/100 g dw		
		Freezing/defrosting	Frozen at −80 °C for 24 h, and then thawed at room temperature for approximately 3 h	13.74 ± 1.23 g/100 g dw		
**Folch Method**	*Lipomyces kononenkoae*	Bead beating	6 cycles of 60 s with 0.5 g of glass beads	−	35.8	[87]
	*Lipomyces starkeyi*	Electroporation technique	Voltage 4 kV, pulse frequency 100 Hz and pulse width 0.01 s with 8 min EP treatment	−	28.84	[125]
	*Yarrowia lipolytica* JMY4086	Bead beating	Vortexed with glass beads for 20 min	24.2 g/L	31	[126]
	*Trichosporon oleaginosus* ATCC20509	Ultrasonic-assisted extraction	Frequency 520 kHz with 40 W of power	−	99.71	[127]
